# Beyond attendance: participation and relational work in planned group recreational activities in a long-term care village setting

**DOI:** 10.1093/geront/gnag211

**Published:** 2026-08-31

**Authors:** Ziying Zhang, Habib Chaudhury, Amy Salmon, Atiya Mahmood, Lillian Hung

**Affiliations:** Department of Gerontology, Simon Fraser University, Vancouver, British Columbia, Canada; Department of Gerontology, Simon Fraser University, Vancouver, British Columbia, Canada; Centre for Advancing Health Outcomes, Providence Research, Providence Health Care, and Faculty of Medicine, University of British Columbia, Vancouver, British Columbia, Canada; School of Population and Public Health, University of British Columbia, Vancouver, British Columbia, Canada; Department of Gerontology, Simon Fraser University, Vancouver, British Columbia, Canada; School of Nursing, University of British Columbia, Vancouver, British Columbia, Canada

**Keywords:** Dementia, Ethnography, Person-centered practice, Meaningful engagement, Supportive relationships

## Abstract

**Background and Objectives:**

Person-centered care (PCC) has shaped dementia care reforms and informed innovative long-term care models such as care villages. Recreational activities are often seen as a site where PCC is enacted, yet little empirical work has examined how participation in structured activities is enabled, mediated, and constrained through everyday social, relational, and organizational dynamics. This study examines how participation in structured group activities was produced in a care village.

**Research Design and Methods:**

An ethnographic study was conducted in Canada’s first publicly funded care village, drawing on 100 hr of participant observation, 12 staff interviews, and informal resident conversations. Data were analyzed using qualitative content analysis.

**Results:**

Participation varied across activity types and residents’ fluctuating comfort, stamina, and readiness. For many residents, participation was not simply an individual decision but was scaffolded through staff invitation, accessible information, relational familiarity, and individualized encouragement. It also depended on logistical and organizational conditions, including preparation, transportation, staffing coordination, and role boundaries. These findings show that participation was relationally and organizationally produced rather than simply a matter of resident preference or program design.

**Discussion and Implications:**

Structured activities can support meaningful engagement when sustained by relational, logistical, and organizational work. The findings clarify how PCC commitments such as choice, autonomy, and inclusion are enacted through activity systems, while highlighting the labor-intensive conditions required for more equitable participation. These insights have implications for staffing, coordination, and activity provision in care villages and long-term care settings.

Structured recreational activities are widely recognized as an important part of the everyday life of residents in long-term care settings, with potential benefits for social connection, emotional well-being, meaningful engagement, and quality of life (Cohen-Mansfield, 2018; Mansbach et al., 2017; Tak et al., 2015). Yet residents engage in these activities in very different ways, shaped by mood, interactional context, interests, and capacities (Beerens et al., 2018; Harmer & Orrell, 2008). These patterns suggest that the key issue is not simply whether activities are available, but how participation in them is made possible in everyday life in care settings.

Existing research has generated important insights into activity provision, resident preferences, participation levels, and the physical, social, and organizational conditions associated with engagement in long-term care (Clarke et al., 2025; Cohen-Mansfield, 2018; Harmer & Orrell, 2008; Lowndes et al., 2021; Plys, 2019; Smit et al., 2014; Stoddart et al., 2024). However, less attention has been paid to how residents are brought into and supported within structured activities. In this view, participation needs to be recognized and understood beyond attendance or preference; it also involves forms of invitation, recognition, coordination, and support that are often left in the background.

This question is especially relevant in innovative long-term care models such as small-household living, clustered domestic models, green care farms, and dementia villages, which seek to move beyond institutional routines by emphasizing domestic scale, autonomy, everyday engagement, and community life (de Boer et al., 2017; Harris et al., 2019; Krier et al., 2023; Shannon et al., 2024). These settings make stronger promises about meaningful activity participation for the residents than traditional long-term care models, yet the literature on them has often focused on physical environment features, implementation, and broad outcomes rather than on the everyday processes and interactions through which such ideals are enacted in practice. Reviews of innovative care models suggest growing interest, but there is limited evaluative and ethnographic evidence, especially regarding how daily routines, staff practices, and care organization shape residents’ actual involvement in communal life and activity engagement (Krier et al., 2023; Sturge, 2024). Innovative settings therefore make it more important to examine how engagement is practically accomplished.

In long-term care settings, structured activities are one avenue through which broader person-centered care commitments are translated into everyday practice. In this study, person-centered care is understood primarily in relation to those dimensions most directly tied to structured activity participation: knowing the person, supporting autonomy and choice, fostering inclusion, and enabling meaningful engagement in everyday life (Fazio et al., 2018; McCormack, 2004). These commitments form an important part of the normative and organizational context of an innovative care setting, such as a care village. At the same time, person-centered care has often been critiqued for being conceptually broad and for paying insufficient attention to the relational, organizational, and structural conditions needed to enact its core commitments in practice (Manthorpe & Samsi, 2016; Rockwell, 2012). Relational approaches are useful as they draw attention to the interpersonal, emotional, and organizational processes through which participation becomes possible, sustained, or constrained in daily life. In models that explicitly seek to reduce institutionalism and foster community life, structured activities are therefore more than program offerings; they are one way in which choice, inclusion, and everyday participation are made visible and enacted. Recent work on clustered domestic models suggests that person-centered environments may create stronger opportunities for engagement, while also indicating that participation in activities still depends heavily on staff who know residents well and can support them in ways that fit their interests and needs (Shannon et al., 2024).

This study explores participation in structured recreational activities in a care village. Rather than treating participation as a matter of individual decision, fixed preference, or simple attendance, it explores the everyday processes through which residents gain access to activities, are brought into participation, and remain involved once they are there. In doing so, it asks how person-centered commitments such as choice, inclusion, and meaningful engagement are enacted in daily routines. It offers a practice-oriented account of structured activity participation in long-term care.

## Research design and methods

### Site description

This research was conducted in Canada’s first care village in the public healthcare system in British Columbia. In this article, a “care village” refers to a dementia village-inspired long-term care model based on the De Hogeweyk care concept developed in the Netherlands. The term reflects the terminology used by the study site. Opened in July 2024, the village accommodates 156 residents across 13 households of 12 residents each, organized into three neighborhoods. Two households were purposively selected for this study because they included residents with varying cognitive and physical support needs. Descriptive information about residents in these two focal households is provided in Table 1. All residents had relocated from a pre-existing long-term care home. During the study year, both households experienced turnover, with capacity maintained through new admissions. Residents in the two focal households had varying cognitive support needs, broadly ranging from moderate to advanced dementia or comparable cognitive impairment, based on staff knowledge, care records, and field observations. The study did not include standardized cognitive assessments or detailed resident-level data on ethnicity, language, cultural background, Indigeneity, or immigration history; these dimensions were therefore not used as bases for formal comparison.

**Table 1:** Demographic information of the two selected households.

Household	Age		Sex (biological classification)		Dementia diagnosis on record	
	Mean	Range	Male	Female	Formal diagnosis recorded	No formal diagnosis recorded
**August 2024**						
** Household 1**	85.5	77–96	6	6	10	2
** Household 2**	86.5	78–97	5	7	7	5
**July 2025**						
** Household 1**	84.1	78–96	5	7	10	2
** Household 2**	85.9	79–90	4	8	6	6

### Data collection

This study employed an ethnographic approach informed by relational and practice-oriented perspectives (Abbott, 2024; Hammersley & Atkinson, 2019), using close observation of everyday practices to examine how participation in recreational activities was shaped by the village’s care philosophy, staffing arrangements, and daily routines. Rather than treating participation as an individual attribute or a simple outcome of activity provision, this orientation directed attention to the everyday practices, relationships, routines, and organizational arrangements through which participation was enabled, sustained, or constrained, including unequal access, role boundaries, and institutional routines. Data were collected over a 12-month period (August 2024–July 2025) using multiple ethnographic methods, including participant observation and semi-structured and informal interviews (Adeoye‐Olatunde & Olenik, 2021; Kvale & Brinkmann, 2009).

Ethical approval for this study was obtained from the Research Ethics Boards at Simon Fraser University (approval no. 30002275) and the University of British Columbia (approval no. H24-02918). The study was approved as a low-risk ethnographic observation in shared care home areas. For residents who might not be able to provide fully informed consent because of cognitive impairment, initial consent was sought from authorized third parties through the care home’s internal communication system. The research team did not have access to family contact information, and residents without third-party consent were not purposefully observed, approached for informal conversation, or included in field notes. Residents’ own assent and dissent were monitored throughout fieldwork.

Observations by the first author took place in the village’s community center and in shared household spaces (dining rooms, living areas, and hallways), avoiding private areas. Observations followed the full trajectory of activities, from invitation and preparation to participation and return. An observer-as-participant stance enabled proximity to these processes without directing them, making visible the relational and logistical work involved in facilitating participation. Four rounds of observation (October and December 2024; March and July 2025) totaled 100 hr and were conducted intermittently between 9:00 a.m. and 8:00 p.m. to align with scheduled group activities. Each fieldwork round lasted approximately five days, with daily observation periods typically lasting around 5 hr, although timing and duration varied according to household routines and scheduled activities. Because structured activities were embedded in broader village life, the fieldwork also captured unstructured engagement, household routines, and general observations of the care environment. For this article, analysis focused on episodes in which structured activity participation or activity-related processes were visible, including information sharing, invitation, preparation, accompaniment, participation, withdrawal, return to households, and staff coordination.

Observed attendance varied by activity type. Large-group activities in the community center typically involved approximately 20 residents from across the village, while smaller therapeutic, interest-based, or social activities generally involved three to ten residents. More intensive follow-through focused on the two 12-resident focal households. Within these households, approximately two residents per household regularly attended structured activities, while another two to three residents per household participated more selectively depending on activity fit, timing, readiness, and available staff support. These indicators convey the scale and variability of observed participation rather than defining a fixed resident sample. Fieldnotes were written on site and expanded daily, informing subsequent observations.

Dialogic data were gathered through semi-structured interviews with staff and informal conversations with residents. A total of 12 staff members were interviewed, including three Recreation and Leisure Aides, two Licensed Practical Nurses, three Health Care Aides, two Multi-Service Workers, and two administrators. Staff were purposively recruited during fieldwork based on their involvement in activity-related routines and variation in roles across the two focal households. Interviews focused on how structured activities were organized and supported, how residents were identified and invited, how participation was facilitated in practice, and what relational, logistical, and organizational factors enabled or constrained engagement. Interviews lasted between 30 and 60 min and were audio-recorded and transcribed verbatim.

Brief field interactions occurred with many residents in the two focal households during everyday routines and activity-related moments. More focused informal conversations about activity experiences occurred with five residents during or immediately after observed activities. These brief interactions explored residents’ immediate experiences, preferences, interests, and broader impressions of daily life in the village. Conversations were guided by residents’ verbal, non-verbal, and affective cues and approached through ongoing process consent. Residents were engaged only when they appeared comfortable and willing to interact; when they appeared fatigued, distressed, uninterested, or unable to sustain the interaction, conversations were not initiated or were discontinued. Residents’ comments were used to contextualize observed participation and inform interpretation of preferences, comfort, and meaning, rather than as formal interview data intended to represent comprehensive resident accounts.

### Data analysis

Data analysis was ongoing and iterative throughout fieldwork. Field notes and transcripts were read repeatedly and analyzed using qualitative content analysis (Elo & Kyngäs, 2008; Graneheim & Lundman, 2004; Mayring, 2014). Meaning units were identified, condensed, and inductively coded, with categories derived from the data. Observations and interviews were coded separately and then compared and integrated. Person-centered care (PCC) later served as a sensitizing concept guiding interpretation, particularly in examining how choice, autonomy, individualized support, and meaningful engagement were enacted in everyday activity practices. Analysis focused on how participation was shaped through everyday practices, including staff roles, routines, and institutional constraints. Coding therefore included attention to exclusion, unequal access, logistical gatekeeping, and tensions between person-centered ideals and everyday constraints.

Rather than treating saturation as a formal endpoint, we assessed analytic sufficiency through the recurrence and explanatory adequacy of patterns across fieldwork rounds, activity types, staff roles, and the two focal households. Later observations reinforced recurring patterns related to invitation, preparation, accompaniment, staff coordination, and the repeated participation of a familiar subgroup, while also refining the analysis by showing that non-attendance could reflect preference for quieter household-based activities or discomfort with group settings, rather than exclusion or disengagement alone. In this sense, later fieldwork primarily deepened and complicated existing interpretations rather than introducing substantially new lines of analysis.

### Trustworthiness and researcher reflexivity

Several strategies enhanced trustworthiness, including triangulation across observations, interviews, and informal conversations; detailed field notes; reflexive memos; an audit trail of analytic decisions; and peer debriefing. Transferability was supported through thick descriptions of the setting and everyday practices (Geertz, 2008). Reflexivity was also central to the study. As a non-staff researcher gradually embedded in daily routines, the first author reflected on how her assumptions about structured activities, her field presence, and residents’ right not to participate shaped data collection and interpretation.

## Results

The findings illustrate how structured activities in the village are organized and experienced in everyday practice. We begin by outlining the activity landscape, including formats, schedules, and staffing support, before examining residents’ varied engagement across activity types. We then turn to the factors shaping participation and the hidden work of access, invitation, and accompaniment that together determine opportunities for involvement.

### Structured activity landscape: forms, schedules, and support structures

Structured activities at the village fell into four broad types—large group activities, small-group therapeutic and interest-based activities, social activities, and outings—which varied in format, facilitation, location, and participant structure (Table 2). Their delivery depended on coordinated staffing support from two types of Recreation and Leisure Aides (RLAs): community-center RLAs (CC RLAs), who primarily facilitated scheduled programs in shared activity spaces, and neighborhood RLAs (NH RLAs), who were based in households and supported residents’ movement, preparation, and participation. These staff worked alongside therapists, volunteers, and community partners.

**Table 2:** Characteristics of structured activities offered at the village.

Activity type	Participants	Content	Led by	Location	Frequency and timing
**Large group**	Approximately 20 residents from across the village	Physical exercise, games, music, movies, holiday celebrations	RLA	Community hall; courtyard (summer)	3 to 4 times per day; morning, afternoon, and evening
**Small-group therapeutic and interest-based activities**	3 to 10 residents, pre-selected (e.g., via referral)	Music therapy, art therapy, church service, stitch ’n time, mental aerobics	Art/music therapists, RLA, faith volunteers	Multi-purpose room, art studio, church	Several per day; varies
**Social**	Approximately 5 residents, informal drop-in	Story time, newspaper reading, games	RLA	Bistro in the community center	Two times per day; morning and midday
**Outing**	Around 10 residents per neighborhood	Scenic drives, shopping, lunches	RLA	Off-site	Monthly per neighborhood; varies

### Varieties of engagement: resident participation across activity types

#### Large group activities

A key feature of large-group activities was the relatively high staff-to-resident ratio. Alongside the CC RLA leading the session, NH RLAs or volunteers assisted residents who could not participate independently. For instance, during bingo, RLAs sat at each table to help mark cards; at a casino night, volunteers managed logistics (e.g., rolling the dice and spinning the wheel) and encouraged participation. This staffing ensured residents with different abilities could engage meaningfully. By contrast, when only one CC RLA led a stretching session, several residents struggled to follow the movements and disengaged.

Engagement in large group activities was diverse, shaped by residents’ individual limits of comfort, ability, and tolerance for activity length. Some residents were actively engaged, for example, by consistently responding to the RLA’s questions, while others preferred to stay in the corner and observe. Throughout the activity, residents typically showed higher levels of attentiveness at the beginning, though their engagement often fluctuated as the session progressed. As the session continued, some residents closed their eyes, while others began scanning the room or shifting their attention away from the facilitator. Activities usually lasted for 1 hr, but some residents left midway or expressed a desire to end early. One resident remarked, “Too long, too much.” Despite such differences, residents generally offered positive feedback when asked about their experiences. Their responses were often brief, using words like “good” or “very fun.” One resident specifically remarked, “It’s good to see old people playing,” while another stated, “I feel good for going.”

Across these observations, large-group activities showed that participation could be enjoyable for some residents yet uneven overall, with engagement depending not only on activity format but also on the level of staffing support and residents’ fluctuating comfort and stamina.

#### Small-group therapeutic and interest-based activities

Within the village’s activity schedule, these smaller programs were operationally grouped with “club” activities, but they included both therapist-led therapeutic sessions and interest-based groups. Therapeutic sessions, such as art and music therapy, were led by trained therapists and often grouped residents by ability or support needs. Interest-based groups, such as book club, Stitch ’n Time, and pen pal activities, were moderated by Recreation and Leisure Aides or volunteers and often arose from residents’ suggestions and evolving interests.

The smaller group format fostered a calm, social environment that supported interpersonal engagement and emotional well-being. At the beginning of music therapy, residents were greeted musically and often responded in song, creating what one therapist called “a sense of togetherness.” Some residents joined in confidently and with ease, while at other moments the therapist moved around the room, singing individual greetings to residents by name. Residents responded in their own ways—by singing back, smiling, making eye contact, or quietly echoing the melody. These activities are also held in quiet, private rooms to minimize distractions, as the activity facilitator noted—“The less distraction, the better”—creating a calming environment in which residents could focus more comfortably on what they wanted to do.

Small-group therapeutic and interest-based activities fostered individual engagement through resident-centered approaches, with therapists encouraging enjoyment over skill. As one therapist explained, “Some people worry about their skills or feel unhappy with their level. I tell them that’s not important—it’s about having fun together. We don’t have to draw; we can look at picture books and chat—that’s great too.” For example, one resident who had stopped painting after a stroke gradually resumed the activity. The therapist reassured him that he did not need to paint the way he used to—only in whatever way felt comfortable to him. Over time, painting appeared to re-enter his routine during the study period.

Beyond therapeutic sessions, these smaller activities also included interest-based groups that were introduced by staff in response to residents expressed preferences during daily interactions and resident meetings. One resident, who often spoke of her love for reading, initially struggled to find suitable materials. After the introduction of a book club, she began carrying her book everywhere and shared enthusiastically, “I can’t remember everything I read, but I’m happy to go and talk.”

Overall, smaller, quieter, and more personalized formats appeared to support more responsive and personally engaging forms of participation, allowing engagement to emerge through familiarity, comfort, and residents’ own interests.

#### Social activities and community outings

Social activities are small group gatherings organized in a relatively flexible format, encouraging relaxed interaction and spontaneous sharing. Typically initiated by the RLA, these sessions feature story time, newspaper reading, and conversational games, with topics including community news, holiday traditions, and personal life. Participation was relatively variable, ranging from as few as two or three residents to more than ten, particularly when sessions followed a large group activity. In smaller groups, RLAs often tentatively introduced a theme, after which residents chatted casually, sometimes digressing into humorous side stories. In larger groups, however, the atmosphere was quieter, as many participants had just come from a large group activity; staff often offered drinks and snacks, and only occasional conversations unfolded among residents.

Outings were arranged monthly for each neighborhood, with each neighborhood comprising four households, and included activities such as scenic drives, shopping trips, or lunches. Participation in these outings provides residents with opportunities to connect with the local community and enjoy time outside the village. On one occasion, residents were taken by bus to buy hamburgers and then shared the meal by the seaside. However, spots are limited due to the small size of the shuttle and the staffing and coordination required to support the outings.

These activities created relatively open and socially engaging forms of participation but also made visible how opportunities beyond the household were shaped by practical limits of staffing, transportation, and coordination.

#### Uneven participation and program fit

These observations indicate that participation was shaped not only by residents’ preferences but also by how activity content aligned with residents’ longstanding interests and familiar ways of life. Staff often remarked that many residents had spent their lives golfing, fishing, or hiking—pastimes tied to the rural coastal context but absent from the schedule—and a few residents themselves recalled these activities when talking about their daily lives. This mismatch also appeared along gendered lines: men were frequently observed sitting back or declining to join, prompting one staff member to suggest that “we need to find a way to stimulate the men more, like a sports night.”

More broadly, observations revealed that a consistent group of residents frequently participated in structured activities. Staff similarly remarked, “it’s always the same ones who come.” Several factors appeared to contribute: residents with more stable health or mood were more able and willing to attend regularly, and staff practices of inviting familiar participants reinforced this recurrence. At the same time, residents requiring higher levels of support were less often visible in group settings.

### Conditions and comfort: moment-to-moment fit in participation

Residents’ momentary emotional and physical condition emerged as one important influence on participation, but not as a stand-alone explanation for whether engagement was sustained. Residents’ comfort, energy, bodily condition, and readiness for group engagement could shift over the course of a day, affecting whether they attended activities they had previously shown interest in. For example, one resident told the researcher in the morning that she wanted to attend an evening sing-along but later remained in the household because, according to staff, she had not slept well the previous night and was feeling unwell. During activities, some residents also became restless or inattentive and needed comfort, redirection, or escorting back to the household. These moments suggest that expressed interest did not automatically translate into sustained participation; engagement depended on the shifting alignment among residents’ momentary readiness, activity fit, environmental demands, and facilitation in the moment.

Some residents also felt insecure or uncomfortable when transitioning from familiar household environments into larger, more public spaces. In one case, a resident showed strong resistance at the entrance of the community hall, yelling, “I can’t handle this; go home!” Another resident asked, “Can I go back? I’m afraid no one knows I am here.” These moments illustrate that willingness alone does not determine participation; the capacity to enter and remain in shared space must be supported emotionally and relationally.

In the village, residents also had greater flexibility to spend time in private (bedroom) or semi-private (household) spaces according to their own preferences. As a result, not attending structured group activities did not always indicate disengagement. Some residents chose to remain in quieter, more familiar settings instead—for example, one resident preferred to knit in her room rather than attend the Stitch ’n Time club, while another declined to go to the community center because it felt “too many people, too noisy.”

### Operationalizing resident choice: staff practices and scaffolding

Respecting choice is embedded in staff’s everyday practice and language. During a team discussion about a resident’s participation status, an RLA reminded the group, “Remember, it’s a choice.” Several staff suggested to the researcher that a resident’s decision not to attend an activity should not be seen as a sign of disengagement or unhappiness. Field observations and informal resident conversations reinforced this interpretation. One resident chose to remain in her room knitting rather than attend the scheduled Stitch ’n Time group. Another resident explained that she did not enjoy large-group settings—“too many people is not for me.” Staff treated both as expressions of resident preference, consistent with the village’s emphasis on honoring choice. As one RLA noted, only those who are interested in the activities will be brought to the community center, and staff avoid bringing residents who are unlikely to benefit. This emphasis on honoring expressed preference reflects the village’s broader philosophy that residents should be supported to direct their own lives.

However, how resident choice is operationalized in everyday life varies considerably. While the village emphasizes freedom of choice, in practice, there are two main ways in which such choice becomes actionable for residents. A small number of residents who were able to articulate preferences and initiate actions independently exercised proactive choice—accessing information, planning, and initiating participation. For the majority of residents, however, choice was scaffolded by staff supports, including informational prompts and interpersonal invitations. The first of these pathways, proactive choice, relied heavily on access to information.

Residents with relatively preserved cognitive function were able to exercise proactive choice, but only when they had access to activity information. For this small group, knowing what was offered and when activities took place provided the essential basis for autonomy—allowing them to read posted schedules, anticipate events, and plan their day independently. Accessibility was shaped by format, visibility, and how staff communicated information in daily routines. Weekly schedules printed on A4 paper were typically posted on walls or held by staff, rarely reaching residents personally. This arrangement could be inconvenient for those wishing to follow the schedule independently. For instance, one resident requested a large-print version; another initially said she “didn’t know what was going on,” but after receiving a printed calendar and having it read aloud, she immediately expressed interest in that evening’s casino night and held onto the page through dinner. Besides paper copies, daily schedules were handwritten on household whiteboards. Some residents responded to these postings, particularly when activities aligned with established interests. For example, one resident, upon seeing the whiteboard, remarked, “Oh, there’s church. I like church. I will go,” though most residents paid little attention to such postings.

Information was conveyed not only through written materials but also informally through staff communication, often during meals or care routines. These interactions created opportunities for residents to receive information and express interest. For example, while assisting with lunch, a care aide casually mentioned the afternoon’s schedule. A nearby resident perked up, asking, “What is that one about?” and then requested, “Call me when it’s time.” Some residents proactively planned their day, others responded in conversation, and many benefited from repeated reminders that increased readiness over time. These formal and informal communications shaped how residents anticipated daily activities. At the same time, only a minority of residents were able to translate such information into participation independently.

For most residents, participation began with being invited. Even if not entirely spontaneous, their decision to attend was still a form of choice—one that was enabled through interpersonal engagement. In one instance, a resident initially declined when a care aide broadly invited several residents to attend an activity. Later, after an RLA greeted him, chatted briefly, and mentioned that a friend of his was already there, he agreed to join.

This invitation-based pathway depended first on how residents were identified as potential participants. Residents were typically identified and invited by staff who believed they might be interested, although invitation practices varied somewhat by activity type. Staff decisions about whom to invite were shaped mainly by relational familiarity, logistical feasibility, and staff initiative. Regular RLAs and care staff, who knew residents’ preferences better, were often more able to identify potentially interested participants. As one care aide explained, “Over time, you start to know which residents might be interested in a particular activity, so you go invite them.”

Participation also depended on whether expressed interest could be followed through in practice. Logistical feasibility often became decisive when residents needed one-on-one support for transportation or participation. As one RLA noted, “Sometimes they want to go, but if no one is available to bring them or stay with them, it just doesn’t happen. I feel bad, because they’ve said they want to go.” In practice, residents requiring more support could not always be considered equally for activities. For example, one resident expressed interest in an activity earlier in the morning while an RLA was updating the household calendar but did not attend when the time came. Although the reason was not entirely clear, the moment suggested that expressed interest still depended on follow-through within staff routines to become actual participation.

Finally, the invitation also depended on how staff approached residents. Some staff tailored invitations to residents’ personal histories and interests, as when one care aide invited a resident to a Girl Scout sing-along during dinner by recalling her past involvement in the organization, prompting a delighted response and immediate agreement to attend. Inviting residents with cognitive impairment also required adapted communication strategies. Staff often adjusted their language and timing to make activities feel familiar, manageable, and personally relevant. As one art therapist advised, “Just say, ‘Your cat (painting) is waiting for you—don’t give him an option.’” Staff also used affirming prompts, reminded residents of prior enjoyment, and, when needed, returned with repeated encouragement. Several staff members observed that if they were the ones to invite a specific resident, that resident was often more likely to accept. In this sense, invitation was not a neutral step preceding participation, but part of the relational work through which participation was made possible.

### Participation under constraint: logistical and organizational infrastructures

Participation was also constrained and enabled by logistical infrastructures and staff coordination, which shaped residents’ ability to move from interest to actual attendance. Although residents might express willingness to attend an activity, participation still depended on substantial preparatory work, coordinated staff support, and organizational follow-through. These conditions were not always consistently available in daily practice.

Once residents agreed to participate, staff often had to complete a series of preparation tasks before escorting them to the community center. Toileting was especially significant. Although public washrooms were available in the community center, residents with more advanced care needs could not be supported there by RLAs and on several occasions had to be transported back to the household when they needed the washroom. Preparation could also involve helping residents put on glasses, dress appropriately for the weather or the activity, and feel ready to leave the household.

Transportation added another layer of complexity. Moving from the household to the community center required navigating elevators, crossing the courtyard, and coordinating escort support, often with one staff member accompanying up to two residents at a time. When more residents wished to attend, staff had to make multiple trips or seek assistance. As a result, even when residents expressed interest, attendance still depended on whether sufficient time and support were available to get them there. These demands sometimes delayed residents’ arrival and disrupted the activity flow. As one art therapist remarked, “It never really happens on time.” At the beginning of group activities, RLAs were often occupied with greeting arrivals, arranging seating, and orienting residents as they entered.

What made these logistical demands especially consequential was that responsibility for supporting participation was not clearly allocated in practice. The village’s staffing structure was designed to promote collaboration between RLAs and care aides in supporting resident engagement. Ideally, care aides managed logistical processes such as personal care and transportation, allowing RLAs to focus on creating engagement opportunities within households. In practice, however, no staff member was officially assigned to handle invitations or transportation, and this ideal often fell short in daily routines. Care aides often initiated invitations, while neighborhood RLAs circulated across households reminding staff about upcoming activities, helping with resident movement, and trying to ensure that interested residents were not overlooked. In the absence of clearly defined responsibilities, this collaborative vision was inconsistently enacted, leaving participation dependent on informal initiative and ad hoc coordination.

Under these conditions, staff intentions to honor residents’ choices could be easily overridden by competing care routines. When households were short-staffed or busy, essential care tasks could displace participation altogether. In one instance, a household skipped sending residents to a morning activity because care aides were occupied with bathing and no one was available to support participation. In another, a family member asked a care aide to reschedule a resident’s evening bath so she could attend a singing activity, explaining that the resident loved singing but rarely expressed this openly. Although the message was passed on, the resident was still brought to the bath and missed the activity. These episodes show that participation depended not only on recognizing residents’ preferences, but also on organizational conditions carrying those preferences through. When communication was fragmented, and routines took priority, even clearly expressed or carefully interpreted choices could be overridden.

## Discussion and implications

This study advances current understandings of structured recreational activities in long-term care in three ways. First, it shows that resident participation in structured activities was more than a matter of program content or preference; rather, it was actively produced through relational, emotional, and logistical support in everyday practice. Second, it reconceptualizes authentic choice not as a discrete moment of individual decision-making, but as a relationally and organizationally supported capacity shaped by familiarity, timing, and access to comprehensible information. Third, it highlights informational, social, and logistical access as critical yet often underrecognized conditions of equitable participation, particularly for residents who require additional support to engage. Together, these findings extend prior work on preference-based recreational activity attendance and resident outcomes (Heid et al., 2025) by showing how preferred or desired activities become actual participation only through everyday systems of information, invitation, preparation, and follow-through. In doing so, the study draws attention to the practical conditions through which person-centered participation is enacted in daily care.

Residents often appeared meaningfully engaged, but this depended on the practical conditions supporting participation (Lowndes et al., 2021). Activities were offered in varied formats and supported by comparatively favorable staffing, while facilitation was often personalized through adjustments in pacing, encouragement, and assistance. At the same time, mismatches in tempo or sensory intensity sometimes led to early withdrawal, underscoring the ongoing work required to align activity demands with residents’ readiness and comfort.

These findings suggest that person-centered participation in structured activities depended on the relational organization of support in everyday practice. Participation depended on how residents were identified, prepared, and supported in the moment. At the same time, structured activities represented only one part of the broader ecology of everyday engagement within the village, which also included more informal, resident-directed, and spontaneous forms of participation embedded in daily routines and interactions. Non-participation in structured activities should therefore not be read too quickly as lack of interest or withdrawal, as it may also reflect mismatch with the activity format, discomfort in larger group settings, fluctuating physical or emotional condition, or preference for quieter and more informal forms of involvement (Plys, 2019). A fuller analysis of these non-structured forms of engagement is beyond the scope of the present paper.

### Reconsidering choice: ambiguity, access, and everyday practice

Resident choice lies at the heart of the village’s activity system, reflecting person-centered care’s commitment to respecting individual preferences, fostering agency, and supporting meaningful involvement on residents’ own terms (Fazio et al., 2018; Kitwood, 1997; McCance & McCormack, 2017). This value was visible in everyday routines: residents were typically invited and consulted before any needed mobility support to take them to group activities, and those who declined were respected. Yet, observed participation patterns and invitation practices raised a critical question—one often left underexamined in person-centered care discourse: what counts as authentic choice? Residents’ consent to take part in any given activity can take place in a variety of ways—planning, responding spontaneously, agreeing after a casual invitation or genuine conversation, or after repeated encouragement. While all of these interactions appeared as “choice,” their implications differed considerably. Some residents consented out of habit or politeness, revealing subtle pressures rather than genuine preference, while repeated encouragement, though often helpful for residents with memory loss, could blur the line between support and implicit coercion. This ambiguity challenges the assumption that verbal agreement invariably signifies authentic interest. Authentic choice was not a discrete decision, but a supported capacity shaped by time, familiarity, and invitation (Wilberforce et al., 2017).

These observations suggest that resident choice was relationally and organizationally co-constructed rather than simply an expression of individual volition. Consistent with relational accounts of autonomy, authentic choice depended on supportive relationships, enabling conditions, and clear, accessible information about what participation entailed (Nedelsky, 1989). In practice, it relied on time and sensitivity to residents’ readiness, as well as familiarity that fostered trust. When these conditions were uneven, verbal agreement could mask uncertainty or subtle pressure, often advantaging residents who were more confident, mobile, or socially fluent.

Although residents were often consulted about activity participation, opportunities for authentic choice were frequently missed, as staff faced practical constraints in identifying and responding to genuine preferences. Authentic choice depends on sustained interpersonal knowledge—familiarity, consistency, and accumulated understanding over time (Brooker & Latham, 2015; Santana et al., 2018). While the village’s care culture emphasizes emotional connectedness, RLAs’ high workloads and limited one-on-one time constrained the development of these relationships. In contrast, smaller household care teams, through daily proximity and deeper familiarity, were often better positioned to notice subtle cues and support meaningful engagement. When structured activities aligned with residents’ interests and capacities, and invitations came from staff who knew them well, participation was more likely to be sustained and positively experienced. These patterns suggest that supported choice can improve the quality of engagement by making participation more personally meaningful and better attuned to residents’ readiness.

The practical realization of choice, shaped by staff familiarity (or lack thereof), resource constraints, and logistical limitations, frequently privileged residents who were more vocal or independently mobile, while those less able to proactively communicate or physically express their interests were more likely to remain peripheral. Residents who initially appeared hesitant or exhibited lower outward enthusiasm often missed opportunities for meaningful social engagement, contributing to a participation pattern that became concentrated among a familiar, active subgroup. As one staff member remarked, “it’s always the same ones who come.” These observations raise important questions about equity in participation, particularly as limited staffing time for accompaniment and relational support may narrow access to structured activities. However, the present study was not designed to systematically examine how these patterns varied across race, culture, language, cognitive status, or other dimensions of difference. Intensive observation was concentrated in two households, and these dimensions were not analyzed comparatively as primary categories. The findings therefore suggest possible inequities in access and support but do not provide a full account of how equity-related factors shaped participation in this setting. These issues warrant more explicit attention in future research.

Our findings also point to an important distinction between supporting residents’ choice about whether to participate and involving residents more directly in shaping what is offered. Resident input in this setting appeared to be expressed mainly through preferences, suggestions, and responses to invitations, rather than through formal partnership in the planning or implementation of recreation (Read et al., 2020; Smebye et al., 2012). This suggests that person-centered recreation may remain largely staff-led in its overall design, even when residents’ preferences are respected at the point of participation (Dupuis et al., 2012; Mondaca et al., 2018).

### Structuring access: information, invitation, and support

Resident participation in structured activities depends not only on expressed interest but also on layered systems of access—informational, social, and logistical—that shape who ultimately engages. Access to understandable information is an important prerequisite for people living with dementia to exercise meaningful choice, not only in care-related decisions but also in everyday activity participation (Hughes et al., 2009; McCabe & Innes, 2013; Swaffer, 2014). In this context, cognitively accessible information about recreational activities is essential if residents are to move from passive acceptance to more active participation. Yet, despite the broader emphasis on accessible information in dementia care, its implementation in relation to everyday activity opportunities remains inconsistent in routine long-term care practice.

Social access—particularly invitations extended by staff—remains uneven, significantly shaped by staff familiarity, time availability, and assumptions about residents’ interests or abilities. As a result, residents who require additional support or express less overt enthusiasm are frequently overlooked, as their engagement needs are harder to interpret or accommodate in the moment.

Invitations tend to be guided by ease of interaction or prior responsiveness, rather than by a deep, evolving understanding of each resident’s preferences and needs (Smebye & Kirkevold, 2013; Tieu & Matthews, 2024). This aspect of staff decision-making has received limited attention in the literature. This gap in the literature may stem from traditional care models, where activities are delivered by designated staff and participation is routinized, leaving little room for discretionary invitations. In contrast, our study—set in a decentralized, socially integrated model—shows how everyday staff decisions about whom to invite shape access to engagement.

Logistical access issues further constrain participation, as the practical supports required—such as helping residents get ready and escorting them to activity spaces—are labor-intensive and frequently disrupted by staffing limitations and environmental factors. These efforts reflect staff’s strong and sustained commitment, yet also reveal that residents’ ability to engage depends not only on willingness, but on whether their support needs align with what the system can consistently provide. While person-centered care literature often frames participation as a matter of individual choice, it rarely accounts for the backstage intention, understanding, and labor required to make that choice actionable. In this sense, equitable participation depends not only on preference or motivation but also on the extent to which logistical supports are available and routinized in everyday care. When such supports are limited, opportunities for engagement tend to cluster around residents who require less assistance to take part.

These findings also raise questions about the dominance of structured group activities as the primary model of recreation in long-term care. While such activities could support meaningful participation (Cohen-Mansfield, 2018), they also relied on substantial organizational work and tended to advantage residents whose support needs, stamina, and communication styles fit more easily within scheduled group formats (Webb, 2023). This suggests that structured group activities should not be treated as the sole or default benchmark of engagement, particularly for residents whose participation is more subtle, fluctuating, or difficult to sustain in public group settings (Kontos et al., 2017).

### Implications and future research

These findings point to several practical implications for supporting structured activity participation in long-term care. Participation relied not only on availability of an activity but also on adequate staffing and clear coordination across invitation, preparation, and escorting, suggesting the need to resource both HCA and RLA roles in ways that reflect this enabling work. These findings also highlight a policy gap: regulations may require activity or activation support but often provide limited guidance on workable staffing ratios or coordination responsibilities (Centers for Medicare & Medicaid Services [CMS], 2023; GKV-Spitzenverband, 2022; Residential Care Regulation, 2009). Supporting authentic choice may also require staff to look beyond residents’ immediate verbal agreement and to draw on relational familiarity, flexible invitations, and attentiveness to subtle expressions of readiness or hesitation. In addition, equitable participation depends on residents having access to understandable and cognitively accessible information about activities, supported through verbal prompting, visual communication, and ongoing staff mediation.

Future research could build on these findings in several directions. First, longitudinal research is needed to examine how participation changes over time. Second, future studies should pay closer attention to residents who may remain peripheral to structured activity systems, particularly among residents with higher support needs. Third, comparative research across care villages, small-household models, and more conventional long-term care settings would help identify how different organizational structures, staffing models, and role boundaries shape opportunities for authentic choice, invitation, and access. Finally, future research should also examine how culturally and linguistically responsive activity systems can support equitable participation for residents whose identities, histories, languages, or cultural preferences may not be fully reflected in existing program designs.

### Limitations

This study offers a situated ethnographic account of structured group activities within one care village. Several limitations should be acknowledged. First, informal interactions with residents were limited in depth and frequency and do not substitute for more systematic resident or family perspectives. Second, the study focused on two purposively selected households within a single care village and therefore may not capture the full range of resident experiences or participation patterns across the wider village or other long-term care settings. Resident-level information on ethnicity, language, cultural background, and related dimensions was limited, and the study was not designed to compare participation systematically across these factors or across varying levels of cognitive and physical support needs. Third, fieldwork was conducted during the village’s first year of operation, a formative period of organizational adjustment and evolving activity systems; the findings may therefore reflect early-stage practices rather than a fully stabilized model. Finally, because observation occurred through intermittent site visits, some fluctuations in staffing, resident readiness, or activity routines may not have been fully captured.

## Conclusion

This study offers a process-oriented understanding of how structured group activities are enacted in a care village setting. Rather than viewing activities as fixed offerings or expressions of resident preference, we use them as a lens to examine how participation is supported, sustained, and at times constrained in daily practice. Our findings show that participation is defined not simply by whether residents attend activities, but by how their presence is meaningfully enabled. Participation emerges as a relational and infrastructural accomplishment—dependent on familiarity, time, coordination, and the work of invitation—rather than an individual choice or intrinsic motivation.

This reframing has important implications for small-scale, person-centered care models. As interest in care villages and small-household models grows, attention must extend beyond activity offerings and the physical environment to the everyday roles, routines, and decision-making processes that shape engagement. Developing and implementing care and support systems that empower staff for this enabling work is essential for sustaining residents’ meaningful participation and enhancing their quality of life.

## Data Availability

This ethnographic study is based on field notes and informal conversations that contain potentially identifiable information. In line with research ethics requirements, these materials cannot be publicly shared. De-identified excerpts may be available from the corresponding author upon reasonable request.

## Analytic methods

All analytic procedures are described in the manuscript; no computational code was used.

## Pre-registration

This study was not preregistered.
